# Supplementary material for: Molecular mechanism of condensin I activation by KIF4A
Source: EMBO J. 2024 Dec 17;44(3):682–704. doi: 10.1038/s44318-024-00340-w (PMC11790958; doi:10.1038/s44318-024-00340-w)
Supplement: Supplementary file 3 — Movie EV2 [file 44318_2024_340_MOESM3_ESM.zip › EVmovie2_legend.docx]

**EV Movie 2: DNA Loop extrusion by human Condensin I** **ΔNCAPG without buffer flow.** Corresponding to Fig. 4D-F.
