# Supplementary figures and images for: Molecular mechanism of condensin I activation by KIF4A
Source: EMBO J. 2024 Dec 17;44(3):682–704. doi: 10.1038/s44318-024-00340-w (PMC11790958; doi:10.1038/s44318-024-00340-w)

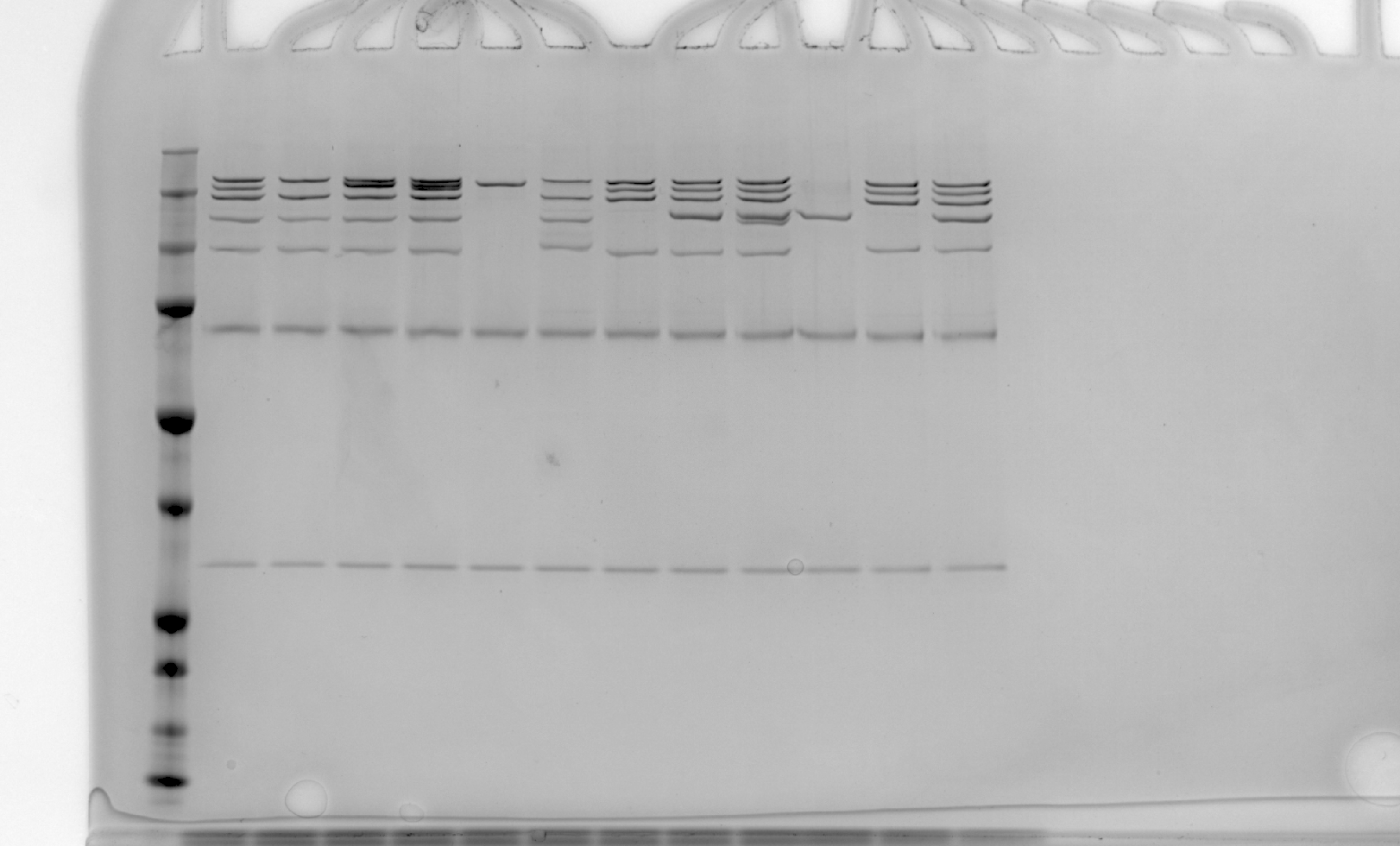

Supplement: Supplementary file 4 — Source data Fig. 1 [file 44318_2024_340_MOESM4_ESM.zip › Figure 1/1B/1b_SDS_gel.tif]

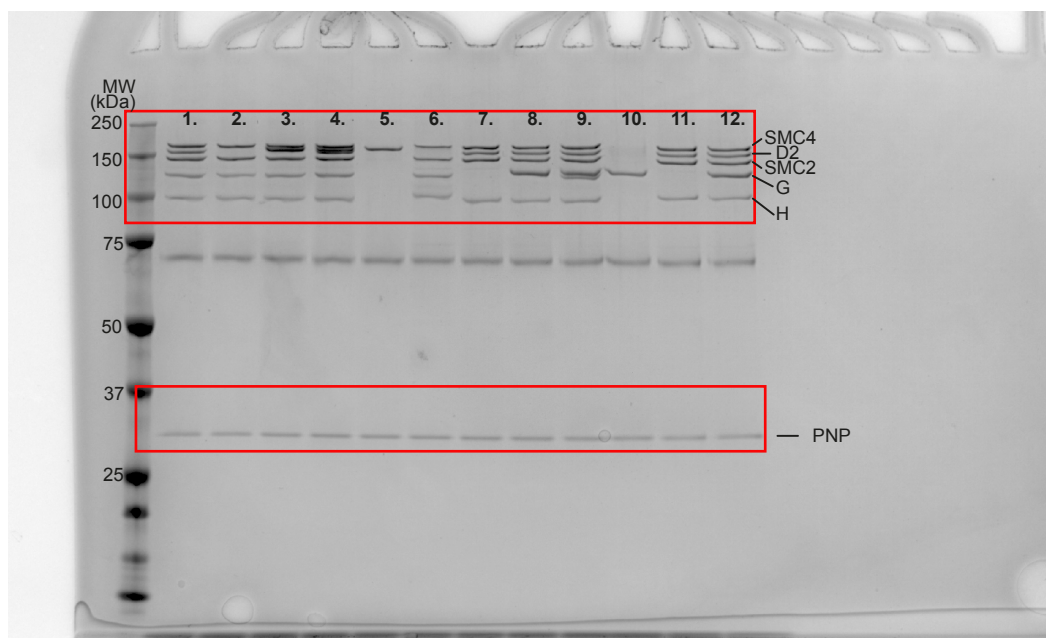

Uncropped Gel from Fig. 1B

Supplement: Supplementary file 4 — Source data Fig. 1 [file 44318_2024_340_MOESM4_ESM.zip › Figure 1/1B/1B_SDS_gel_crop.pdf]

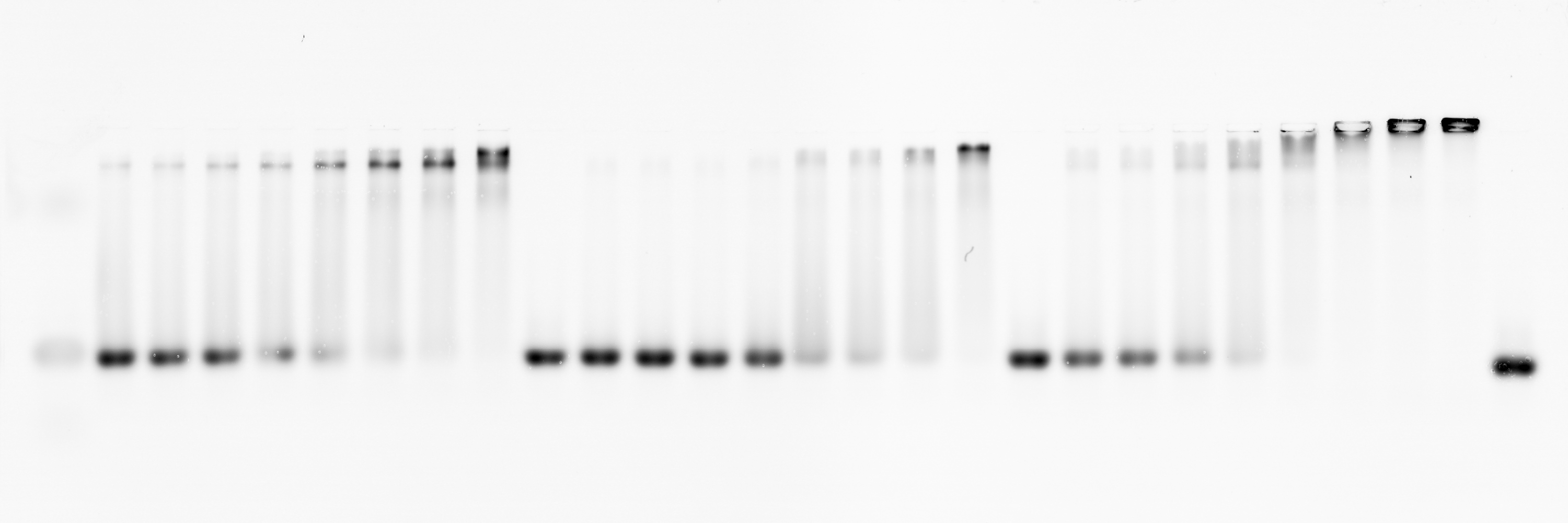

Supplement: Supplementary file 4 — Source data Fig. 1 [file 44318_2024_340_MOESM4_ESM.zip › Figure 1/1D/1D_emsa_CItet_50bp.tif]

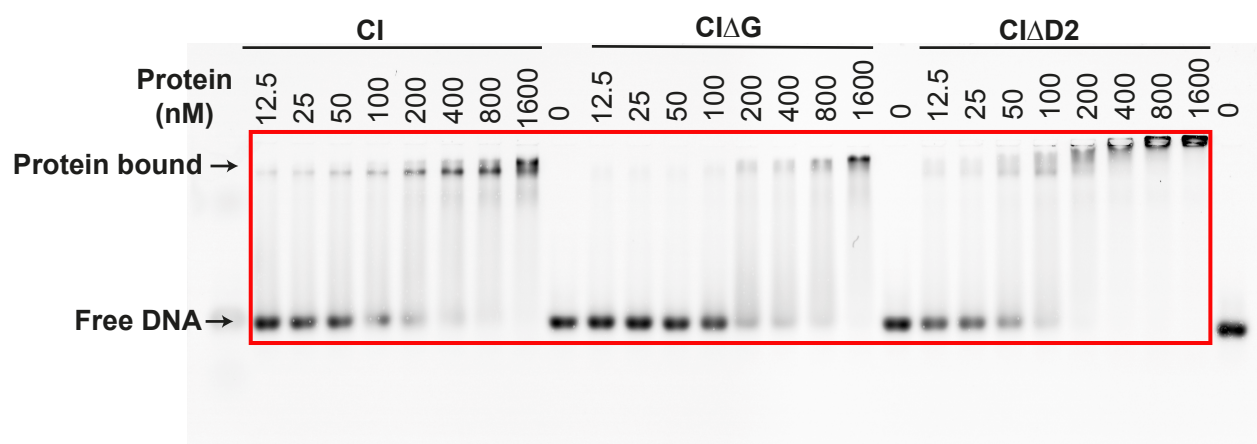

Supplement: Supplementary file 4 — Source data Fig. 1 [file 44318_2024_340_MOESM4_ESM.zip › Figure 1/1D/1D_emsa_CItet_50bp_crop.pdf]

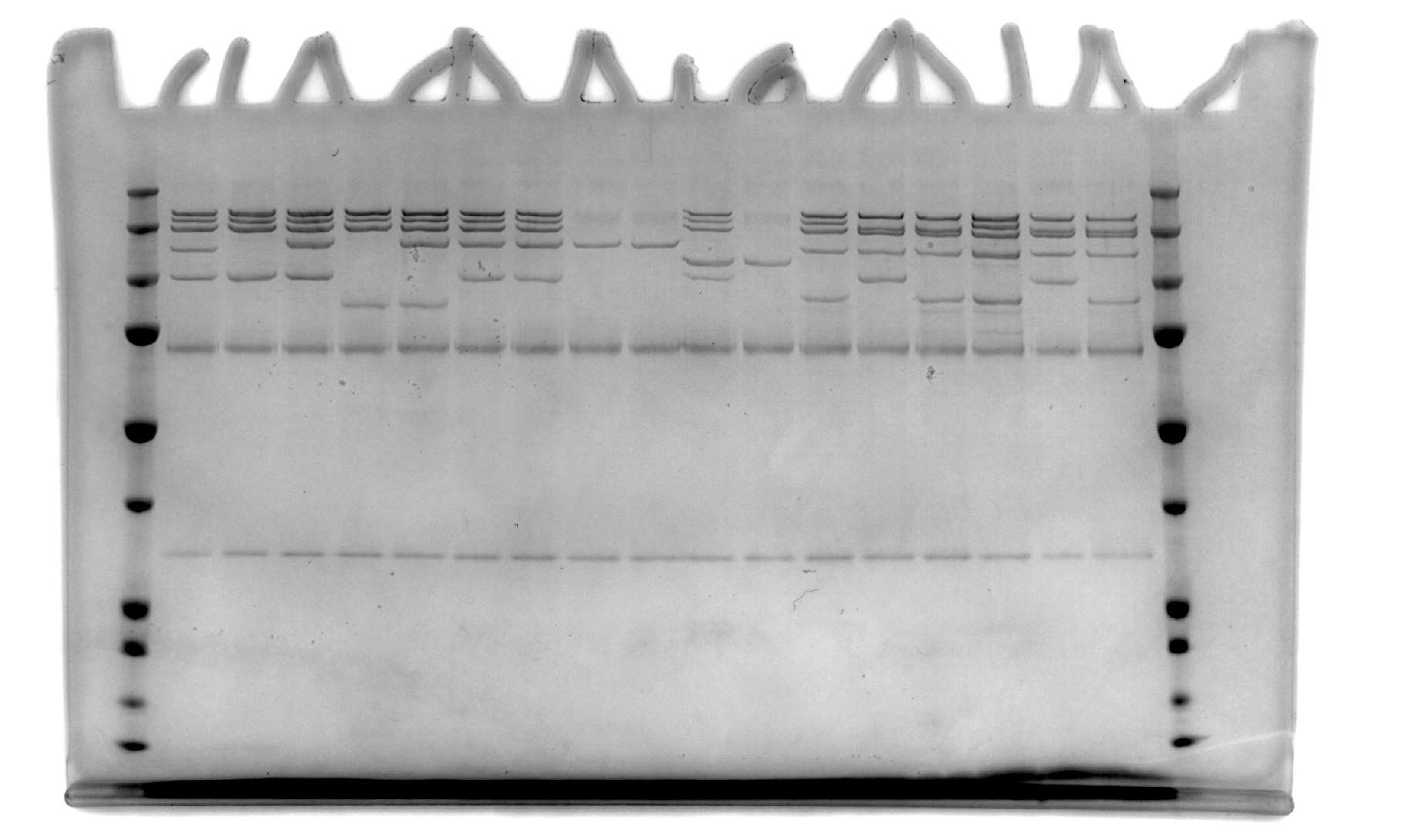

Supplement: Supplementary file 5 — Source data Fig. 2 [file 44318_2024_340_MOESM5_ESM.zip › Figure 2/2A/2a_SDS_gel.tif]

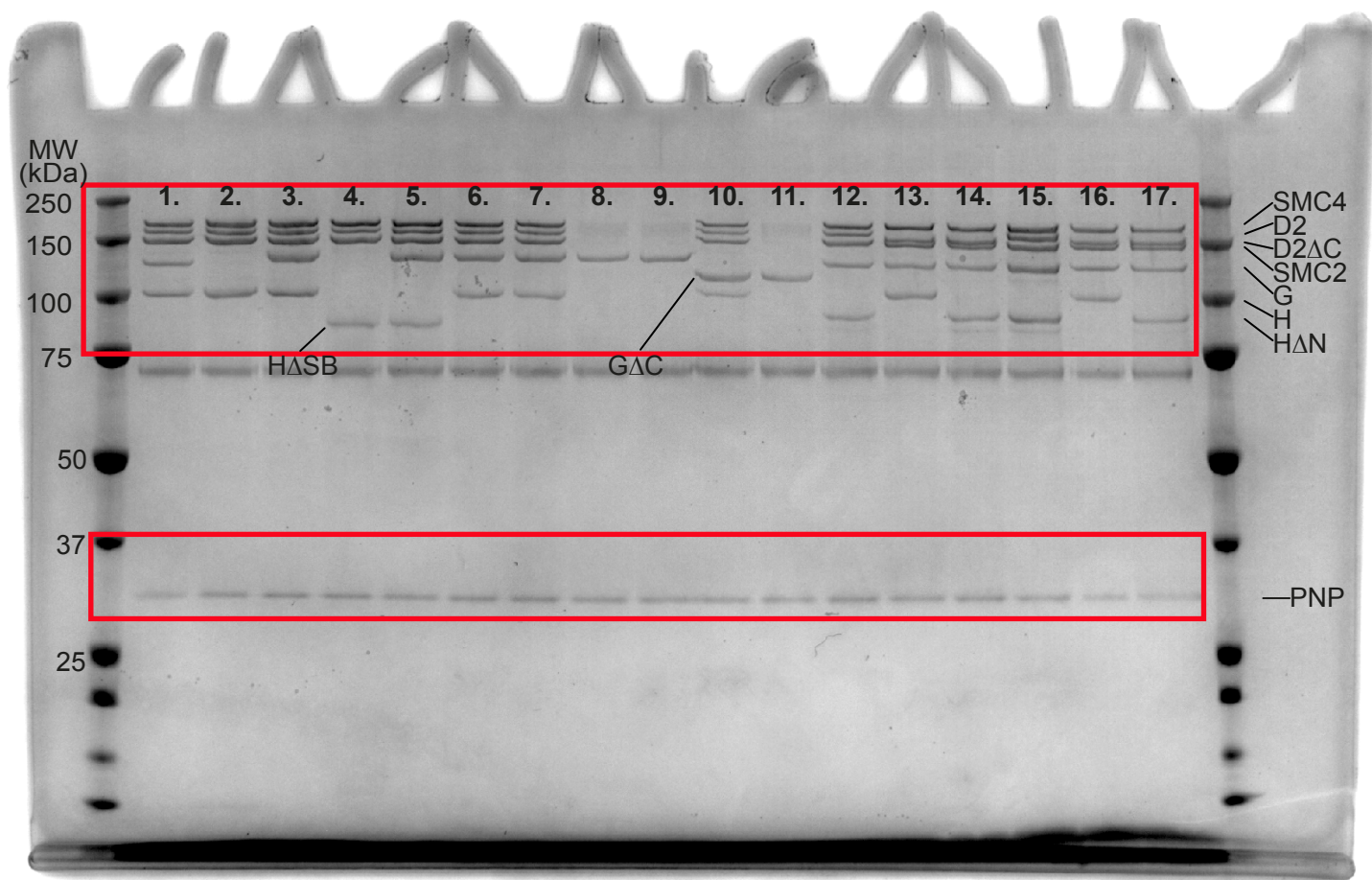

Supplement: Supplementary file 5 — Source data Fig. 2 [file 44318_2024_340_MOESM5_ESM.zip › Figure 2/2A/2a_SDS_gel_crop.pdf]

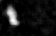

Supplement: Supplementary file 7 — Source data Fig. 4 [file 44318_2024_340_MOESM7_ESM.zip › Figure 4/4A/Fig4a_loopflow_imgs.tif]

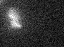

Supplement: Supplementary file 7 — Source data Fig. 4 [file 44318_2024_340_MOESM7_ESM.zip › Figure 4/4A/Fig4a_loopflow_raw_imgs.tif]

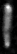

Supplement: Supplementary file 7 — Source data Fig. 4 [file 44318_2024_340_MOESM7_ESM.zip › Figure 4/4D/Fig4d_loopnoflow_imgs.tif]

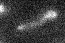

Supplement: Supplementary file 7 — Source data Fig. 4 [file 44318_2024_340_MOESM7_ESM.zip › Figure 4/4D/Fig4d_loopnoflow_raw_imgs.tif]
