# Supplementary material for: Molecular mechanism of condensin I activation by KIF4A
Source: EMBO J. 2024 Dec 17;44(3):682–704. doi: 10.1038/s44318-024-00340-w (PMC11790958; doi:10.1038/s44318-024-00340-w)
Supplement: Supplementary file 10 — Expanded View Figures [file 44318_2024_340_MOESM10_ESM.pdf]

Expanded View Figures

Figure EV1. Condensin I mutant complex quality control.

(**A**) Comparison of two different batches of recombinant CIWT pentamer, and CIΔG and CIΔD2 tetramers. Trend of tetrameric complexes being significantly more active than pentameric condensin I is consistent. Data from three technical replicates. Error bars represent s.e.m. *P* values indicated are from unpaired, two-tailed t test with Welch's correction. (**B**) Mass photometry data of condensin I tetramers, CIΔG, with and without NCAPG being added to reconstitute the pentamer. (**C**) Mass photometry data of condensin I tetramers, CIΔD2, with and without NCAPD2 being added to reconstitute the pentamer. (**D–F**) Mass photometry data of condensin I pentamers, CID2ΔC, CIHΔN and CIHΔN,D2ΔC, respectively, consistent with pentameric mass and confirming they are the major species present. Source data are available online for this figure.

© The Author(s)

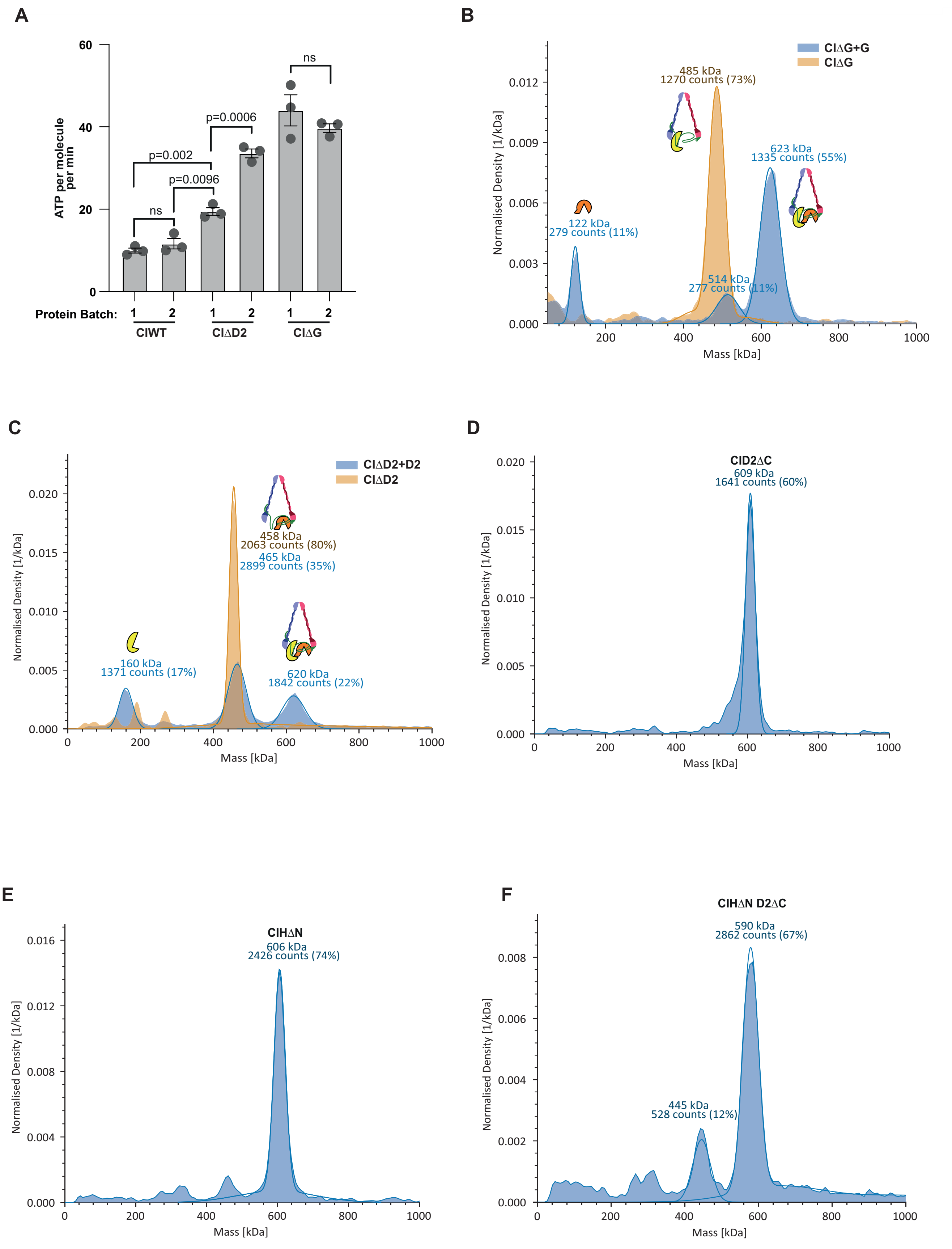
A
ATP per molecule per min
Protein Batch:
CIWT
CIΔD2
CIΔG
ns
p=0.002
p=0.0096
p=0.0006
B
CIΔG+G
CIΔG
485 kDa
1270 counts (73%)
623 kDa
1335 counts (55%)
122 kDa
279 counts (11%)
514 kDa
277 counts (11%)
Normalised Density [1/kDa]
Mass [kDa]
C
CIΔD2+D2
CIΔD2
458 kDa
2063 counts (80%)
465 kDa
2899 counts (35%)
160 kDa
1371 counts (17%)
620 kDa
1842 counts (22%)
D
CID2ΔC
609 kDa
1641 counts (60%)
E
CIHΔN
606 kDa
2426 counts (74%)
F
CIHΔN D2ΔC
590 kDa
2862 counts (67%)
445 kDa
528 counts (12%)

 © The Author(s)

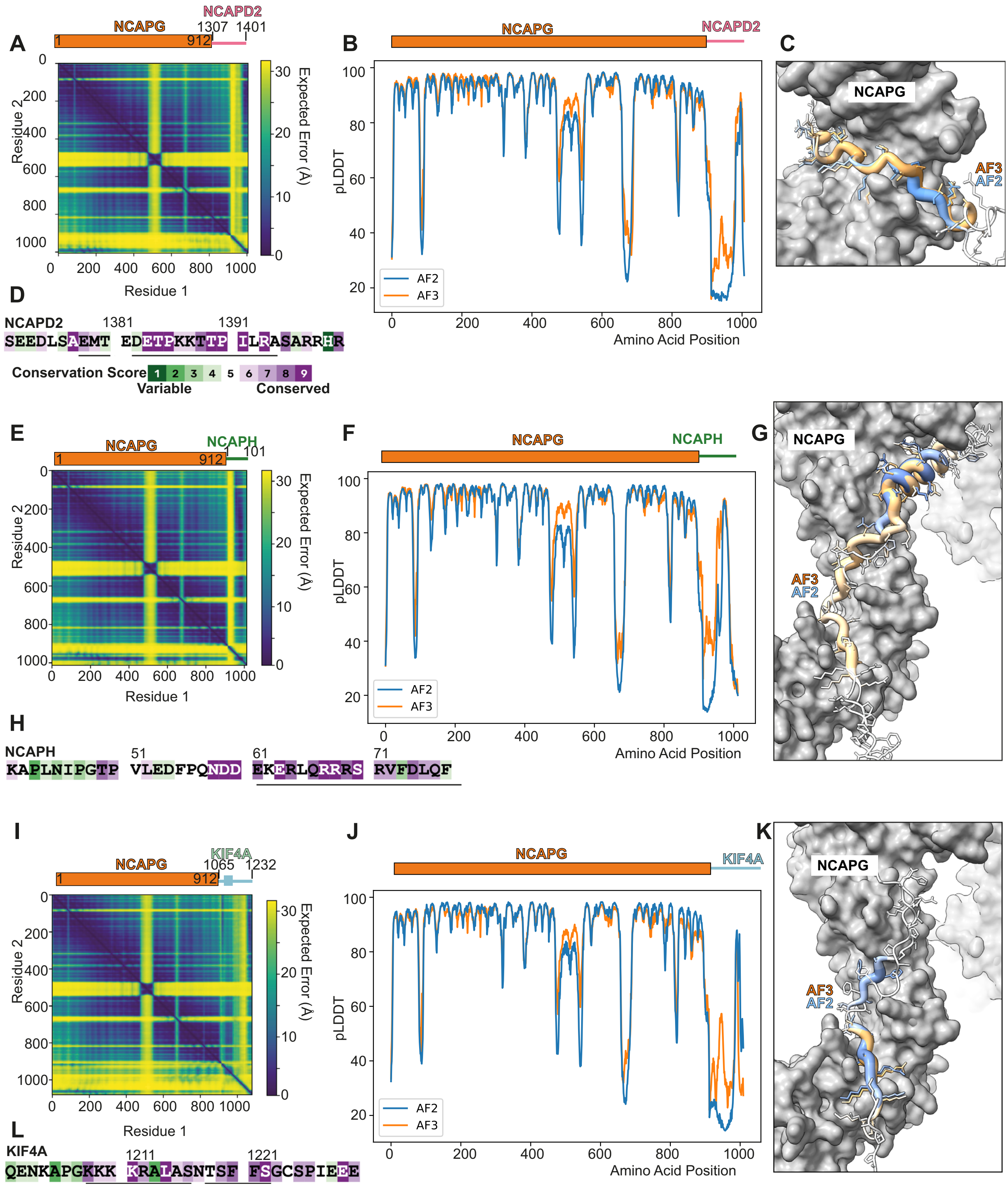

Figure EV2. NCAPG SLiM interaction AlphaFold2 and 3 confidence.

(**A**) AlphaFold2 predicted alignment error plot for interaction between NCAPG and NCAPD2$_{1307\text{-}1401}$. (**B**) Predicted local distance difference test (pLDDT) score per residue of AlphaFold2 and 3 (AF2 and AF3, respectively) predictions of NCAPG and NCAPD2$_{1307\text{-}1401}$. (**C**) Comparison of AF2 (blue) and AF3 (orange) predictions of NCAPG and NCAPD2$_{1307\text{-}1401}$. AlphaFold pLDDT score is represented in thickness of NCAPD2 backbone and colour intensity. (**D**) SLiM region with conservation colouring from ConSurf (Ashkenazy et al, 2016). (**E–H**) Equivalent data to (**A–D**) for prediction of NCAPG with NCAPH$_{1\text{-}101}$. (**I**) AlphaFold2 predicted alignment error plot for interaction between NCAPG and KIF4A$_{1065\text{-}1401}$. (**J–L**) Equivalent data to (**B–D**) for prediction of NCAPG with KIF4A$_{1136\text{-}1232}$.

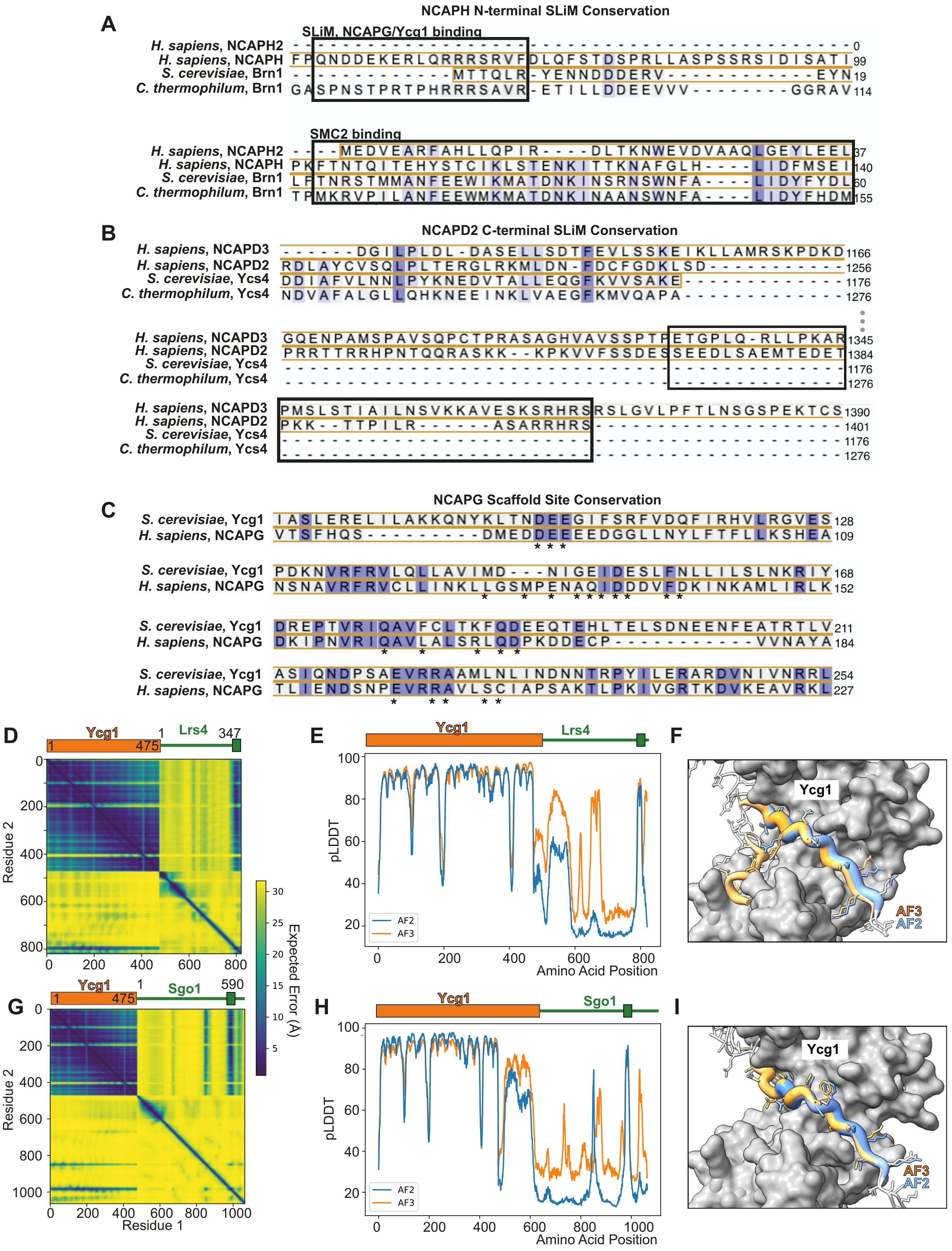
A
NCAPH N-terminal SLiM Conservation
SLiM, NCAPG/Ycg1 binding
H. sapiens, NCAPH2
H. sapiens, NCAPH FPQNDDEKERLQRRRSRVFDLQFSTDSPRLLASPSSRSIDISATI 99
S. cerevisiae, Brn1 --------------MTTQLR-YENNDDDERV-----------EYN 19
C. thermophilum, Brn1 GASPNSTPRTPHRRRSAVR-ETILLDDEEVVV--------GGRAV 114
SMC2 binding
H. sapiens, NCAPH2 --MEDVEARFAHLLQPIR---DLTKNWEVDVAAQLGEYLEEL 37
H. sapiens, NCAPH PKFTNTQITEHYSTCIKLSTENKITTKNAFGLH----LIDFMSEI 140
S. cerevisiae, Brn1 LFTNRSTMMANFEEWIKMATDNKINSRNSWNFA----LIDYFYDL 60
C. thermophilum, Brn1 TPMKRVPILANFEEWMKMATDNKINAANSWNFA----LIDYFHDM 155
B
NCAPD2 C-terminal SLiM Conservation
H. sapiens, NCAPD3 ------DGILPLDL-DASELLSDTFEVLSSKEIKLLAMRSKPDKD 1166
H. sapiens, NCAPD2 RDLAYCVSQLPLTERGLRKMLDN-FDCFGDKLSD 1256
S. cerevisiae, Ycs4 DDIAFVLNNLPYKNEDVTALLEQGFKVVSAKE 1176
C. thermophilum, Ycs4 NDVAFALGLLQHKNEEINKLVAEGFKMVQAPA 1276
H. sapiens, NCAPD3 GQENPAMSPAVSQPCTPRASAGHVAVSSPTPETGPLQ-RLLPKAR 1345
H. sapiens, NCAPD2 PRRTTRRHPNTQQRASKK--KPKVVFSSDESSEEDLSAEMTEDET 1384
S. cerevisiae, Ycs4 1176
C. thermophilum, Ycs4 1276
H. sapiens, NCAPD3 PMSLSTIAILNSVKKAVESKSRHRSRSLGVLPFTLNSGSPEKTCS 1390
H. sapiens, NCAPD2 PKK--TTPILR-----ASARRHRS 1401
S. cerevisiae, Ycs4 1176
C. thermophilum, Ycs4 1276
C
NCAPG Scaffold Site Conservation
S. cerevisiae, Ycg1 IASLERELILAKKQNYKLTNDEEGIFSRFVDQFIRHVLRGVES 128
H. sapiens, NCAPG VTSFHQS---------DMEDDEEEDGGLLNYLFTFLLKSHEA 109
S. cerevisiae, Ycg1 PDKNVRFRVLQLLAVIMD---NIGEIDESLFNLLILSLNKRIY 168
H. sapiens, NCAPG NSNAVRFRVCLLINKLLGSMPENAQIDDDVFDKINKAMLIRLK 152
S. cerevisiae, Ycg1 DREPTVRIQAVFCLTKFQDEEQTEHLTELSDNEENFEATRTLV 211
H. sapiens, NCAPG DKIPNVRIQAVLALSRLQDPKDDECP-----------VVNAYA 184
S. cerevisiae, Ycg1 ASIQNDPSAEVRRAAMLNLINDNNTRPYILERARDVNIVNRRL 254
H. sapiens, NCAPG TLIENDSNPEVRRAVLSCIAPSAKTLPKIVGRTKDVKEAVRKL 227
D
Ycg1 1 475
Lrs4 1 347
Residue 2
Expected Error (Å)
E
Ycg1 Lrs4
pLDDT
Amino Acid Position
AF2
AF3
F
Ycg1
AF3
AF2
G
Ycg1 1 475
Sgo1 1 590
Residue 2
Residue 1
H
Ycg1 Sgo1
pLDDT
Amino Acid Position
AF2
AF3
I
Ycg1
AF3
AF2

 © The Author(s)

Figure EV3. Condensin disordered sequence conservation.

(**A**) Alignment of N-terminal region of NCAPH/H2 and Brn1, in *H. sapiens, S. cerevisiae* and *C. thermophilium*, with NCAPG/Ycg1 and SMC2 binding regions indicated. (**B**) Alignment of C-terminal region of NCAPD2/3 and Ycs4 in *H. sapiens, S. cerevisiae* and *C. thermophilium*. (**C**) Alignment of SLiM docking region in NCAPG and Ycg1, from *H. sapiens and S. cerevisiae*, (*) indicates residues with in 4 Å of predicted bound SLiM. (**D**) AlphaFold2 predicted alignment error of $Ycg1_{1-475}$ and Lrs4. (**E**) Predicted local distance difference test (pLDDT) score per residue of AlphaFold2 and 3 (AF2 and AF3, respectively) predictions of $Ycg1_{1-475}$ and Lrs4. (**F**) Comparison of AF2 (blue) and AF3 (orange) predictions of $Ycg1_{1-475}$ and Lrs4. AlphaFold pLDDT score is represented in thickness of Lrs4 backbone and colour intensity. (**G–I**) Equivalent data as (**D–F**) for $Ycg1_{1-475}$ and Sgo1. Sequence data sourced from Uniprot (Bateman et al, 2023).

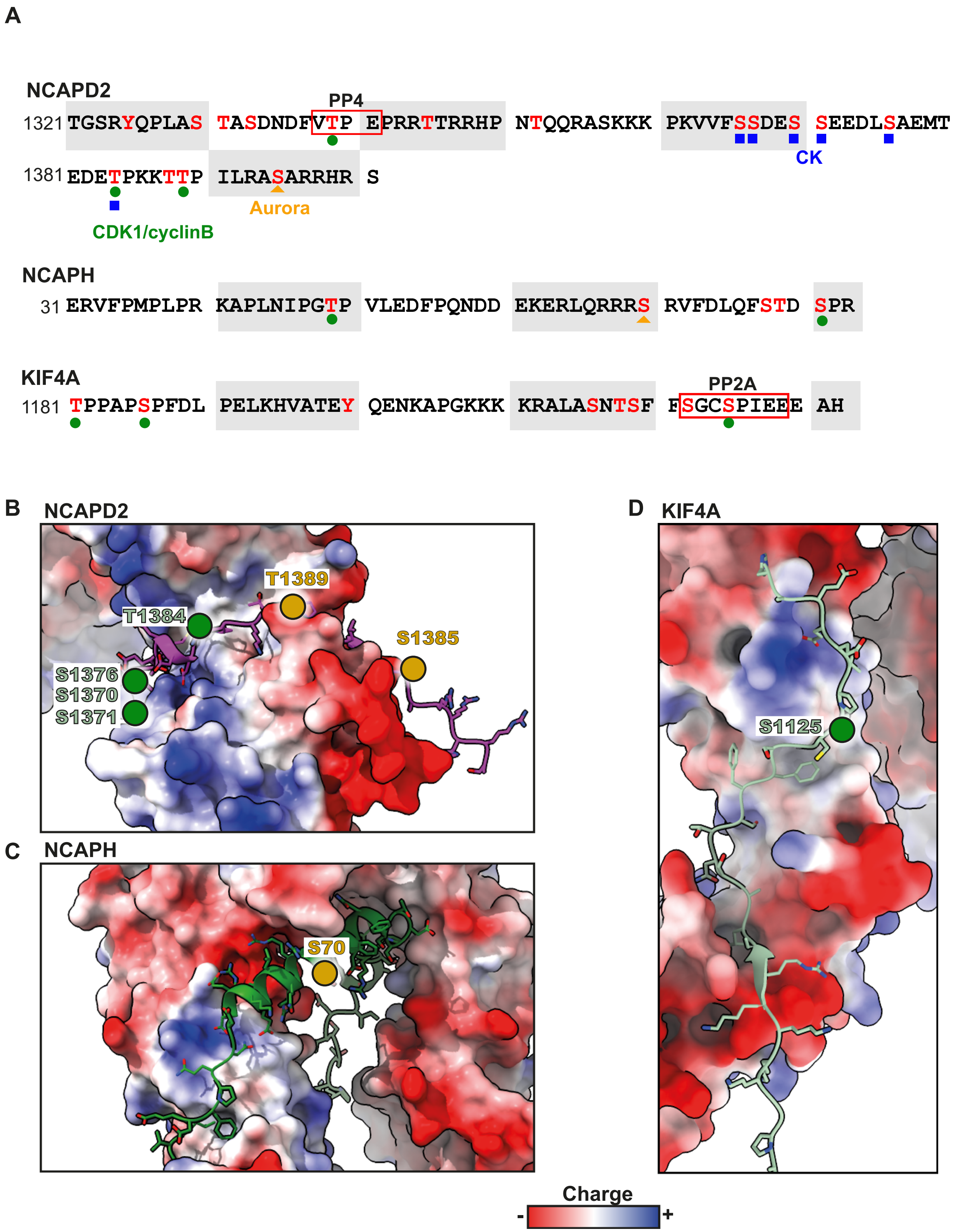
A
NCAPD2
PP4
1321 TGSRYQPLAS TASDNDFVTP EPRRTTRRHP NTQQRASKKK PKVVFSSDES SEEDLSAEMT
CK
1381 EDETPKKTTP ILRASARRHR S
Aurora
CDK1/cyclinB
NCAPH
31 ERVFPMPLPR KAPLNIPGTP VLEDFPQNDD EKERLQRRRS RVFDLQFSTD SPR
KIF4A
PP2A
1181 TPPAPSPFDL PELKHVATEY QENKAPGKKK KRALASNTSF FSGCSPIEEE AH
B NCAPD2
T1389
T1384
S1385
S1376
S1370
S1371
C NCAPH
S70
D KIF4A
S1125
Charge
-
+

© The Author(s)

Figure EV4. Phosphorylation and phosphatase sites in SLiMs.

(**A**) Theoretical sites of phosphorylation by Aurora (orange triangle), CDK1/cyclin B (green circle) and casein (blue square) kinases in SLiM of NCAPD2, NCAPH and KIF4A. Red rectangles indicate phosphatase binding sites. (**B–D**) The location of phosphorylation sites indicated in (**A**) in NCAPD2, NCAPH and KIF4A, respectively. Based off of electrostatic surface charge, the effect of the phosphorylation is coloured either green, for potentially enhancing the interaction or orange, for resulting in a potential charge clash.
